# Translation, Cross-Cultural Adaptation, and Psychometric Validation of Dietary Behavior Instruments into Arabic for the MENA Region: A Systematic Review from BRIDGE Project

**DOI:** 10.3390/nu18142343

**Published:** 2026-07-16

**Authors:** Moncef Maiouak, Sandokane Hounnoukon Noussissy, Marie Claire Chamieh, Soraya Laraqui Houssaini, Imane El Faziki, Faten Abu Najem, Sara Nasr, Soumaya Benmaamar, Ibtissam El Harch, Samira El Fakir, Nada Otmani, Klaus Bös, Laura Wolbring, Mohamed Aly, Osama Abdelkarim, Karima El Rhazi

**Affiliations:** 1 Faculty of Medicine, Pharmacy and Dentistry of Fes, Sidi Mohamed Ben Abdellah University, Fez 30000, Morocco; sandokane.hounnoukonnoussissy@usmba.ac.ma (S.H.N.); soraya.laraqui@usmba.ac.ma (S.L.H.); imane.elfaziki@usmba.ac.ma (I.E.F.); soumaya.benmaamar@usmba.ac.ma (S.B.); ibtissam.elharch@usmba.ac.ma (I.E.H.); samira.elfakir@usmba.ac.ma (S.E.F.); nada.otmani@usmba.ac.ma (N.O.); karima.elrhazi@usmba.ac.ma (K.E.R.); 2 Hassan II University Hospital of Fez, Fez 30050, Morocco; 3 Department of Nutrition and Food Sciences, Faculty of Agricultural and Food Sciences, American University of Beirut, Beirut 1107 2020, Lebanon; mc31@aub.edu.lb (M.C.C.); fia15@mail.aub.edu (F.A.N.); ssn28@mail.aub.edu (S.N.); 4 Institute of Sports and Sports Science, Karlsruhe Institute of Technology, 76131 Karlsruhe, Germany; klaus.boes@kit.edu (K.B.); laura.wolbring@kit.edu (L.W.); osamahalim@ymail.com (O.A.); 5 Faculty of Liberal Arts and Sciences, Chukyo University, Nagoya 461-8710, Japan; mohamed.aly@aun.edu.eg; 6 Faculty of Sport Sciences, Assiut University, Assiut 71515, Egypt; 7 Department of Sports Management, School of Business, ESLSCA University, Giza 12676, Egypt

**Keywords:** dietary, behavior, instruments, cross-cultural adaptation, psychometric validation, translation, Arabic

## Abstract

**Background/Objectives**: Accurate assessment of dietary behaviors is essential for understanding their impact on health and guiding nutritional policies. Given the escalating prevalence of noncommunicable diseases in the MENA region, the availability of culturally adapted and psychometrically validated Arabic-language instruments is essential for accurate health assessment. This systematic review aims to identify and evaluate studies that have translated, adapted, or validated dietary behavior measurement instruments into Arabic. **Methods**: Following the PRISMA guidelines and registered in PROSPERO (CRD420251157552), a search was conducted in PubMed, Scopus, and Web of Science. Studies including the validation, translation, or adaptation of instruments into Arabic among Arabic-speaking populations were included. Methodological quality was assessed using the COSMIN checklist. **Results**: Thirty-two studies were included, published between 2006 and 2025, primarily in Lebanon (*n* = 14) and Saudi Arabia (*n* = 6). The instruments measured various concepts: dietary intake, diet quality, eating disorders, etc. While internal consistency and structural validity were frequently assessed with adequate methodological quality, content validity, measurement error, and responsiveness were either under-reported or failed to meet COSMIN standards. **Conclusions**: Despite an increasing volume of publications, the methodological quality of Arabic instrument validation remains heterogeneous, with significant gaps in the assessment of longitudinal properties. Efforts are needed to improve the rigor of adaptation processes and to evaluate key properties such as responsiveness in order to ensure reliable tools for research and clinical practice in the MENA region.

## 1. Introduction

While diet is fundamentally defined by individual consumption, the specific parameters of a “healthy diet” are context-dependent, shaped by cultural and economic factors. Although undernutrition continues to be a major issue in some regions, the increasing availability of processed foods and unhealthy dietary choices has led to a growing burden of noncommunicable diseases associated with poor nutritional practices [1,2].

Accurately understanding and anticipating the burden of chronic diseases linked to dietary behavior carries important implications for public health planning and intervention. In fact, according to the World Health Organization (WHO), reducing the intake of salt, sugars, saturated fats, and industrially produced trans-fats is essential for a healthy diet, which in turn helps protect against numerous noncommunicable diseases, including diabetes and cancer [3].

As a result, an unhealthy diet represents a major risk factor for numerous chronic diseases, including cardiovascular diseases, cancer, diabetes, and other obesity-related conditions. The Middle East and North Africa (MENA) region faces a high and rising burden of obesity and noncommunicable diseases, driven in part by shifts toward Westernized dietary patterns [4]. Reliable assessment of dietary behaviors is therefore essential for designing effective prevention and early intervention strategies [5,6]. However, a critical methodological gap remains: the scarcity of culturally adapted and psychometrically validated Arabic-language instruments for measuring dietary behaviors in MENA populations.

Accurate assessment of dietary intake is essential for understanding the effects of diet on human health and disease, as well as for informing nutrition policies and the development of dietary recommendations for individuals, groups, and communities [7].

Although numerous diet-related questionnaires and assessment scales have been developed and are widely used at the international level, including well-known tools such as Food Frequency Questionnaires (FFQs) and 24 h dietary recalls (24HRs), there remains a significant shortage of instruments that are both valid and reliable for accurately assessing dietary behaviors in Arabic-speaking populations. This methodological scarcity hinders the collection of harmonized, culturally nuanced dietary data, thereby limiting cross-study comparability and evidence-based practice. Consequently, researchers targeting Arabic-speaking populations frequently adapt existing instruments; however, a rigorous validation process is essential to maintain the original measurement properties within a new cultural context [6,8,9].

Currently, there is a notable lack of a comprehensive synthesis of the existing evidence regarding both the availability and the quality of measurement instruments that have been culturally adapted for use in Arabic-speaking populations. This gap in the literature may contribute to the continued use of tools that have not undergone rigorous validation or reliability testing, which in turn can result in inaccurate data collection, reduced comparability of findings across different studies, and ultimately less effective assessment and intervention in both clinical practice and research contexts. Given the growing importance of accurately assessing dietary behaviors to inform public health policies and interventions, addressing this gap is particularly critical.

Therefore, in response to this need, our systematic review objective is to identify, evaluate, and synthesize all studies that have undertaken the translation, cultural adaptation, and/or validation of Arabic-language instruments designed to measure dietary behaviors in Arab countries, with the aim of providing a clear overview of the current state of available tools and their psychometric properties, thereby guiding researchers and practitioners toward the use of more valid and reliable measures in this context.

## 2. Materials and Methods

### 2.1. Protocol and Registration

At the time of protocol submission to PROSPERO (PROSPERO 2026 CRD420251157552) on 25 February 2026, a search of the registry confirmed that no systematic reviews with identical objectives had been previously registered. We note that the review process began in August 2025. We registered this systematic review protocol in PROSPERO. This review adheres to the 2020 Preferred Reporting Items for Systematic Reviews (PRISMA) guidelines (Supplementary Materials).

This systematic review was guided by a research question structured according to the PICO(S) framework:

- Population (P): Arabic-speaking participants from Arab countries in the MENA region (general population, community settings, or specific healthy groups).
- Intervention/instrument (I): Any instrument (scale, questionnaire, survey, or tool) measuring dietary behavior, eating behavior, or food habits that has been translated, cross-culturally adapted, and/or psychometrically validated into Arabic.
- Comparator (C): Not applicable for the primary descriptive synthesis; however, for hypothesis testing of construct validity, comparisons with the original language version or other validated instruments were considered when reported.
- Outcome (O): Psychometric properties (internal consistency, reliability, structural validity, construct validity, criterion validity, responsiveness, floor/ceiling effects, and acceptability) and the quality of translation/cultural adaptation procedures.
- Study design (S): Cross-sectional validation studies, or studies reporting translation/cultural adaptation procedures.

The formal research question therefore is as follows: What is the quality of translation, cross-cultural adaptation, and psychometric validation of Arabic-language instruments measuring dietary behavior (excluding nutritional knowledge, attitudes, beliefs, or biochemical status) among Arabic-speaking populations in the MENA region?

### 2.2. Eligibility Criteria

#### 2.2.1. Inclusion Criteria

Studies were included if they met the following criteria:

- Population: Arabic-speaking participants from Arab countries in any MENA region country, general population, community setting, or specific healthy groups.
- Instrument: Instruments measuring any facet of dietary behavior, eating behavior, or food habits that have been translated, cross-culturally adapted, or validated in Arabic.
- Type of study: methodological, cross-sectional, or validation studies, including translation/cultural adaptation procedures.
- Language of publication: While no initial language restrictions were applied to the search, only records featuring an English abstract were eligible for full-text assessment.
- Type of publication: Full-text articles.

#### 2.2.2. Exclusion Criteria

We excluded the following:

- Studies using dietary behavior tools that had not been cross-cultural adapted or validated in Arabic.
- Studies not conducted in an Arab country or with an Arab population.
- Instruments measuring only nutritional knowledge, food safety, attitudes/beliefs without behavior, or purely biochemical nutritional status.
- Systematic reviews, commentaries, editorials, opinion pieces, or conference abstracts.
- Studies that only use an Arabic instrument without reporting on any of its translation, adaptation, or validation properties.

### 2.3. Information Sources and Search Strategy

We conducted, on 16 August 2025, an exhaustive search for full-text articles in databases, namely in PubMed, Scopus, and in Web of sciences, without any date restrictions. We used the keywords and combined MeSH terms such as the following:

PubMed/MEDLINE: ((“Validation Studies as Topic”[MeSH] OR “Psychometrics”[MeSH] OR “Cross-Cultural Comparison”[MeSH] OR “Translating”[MeSH] OR (validat*[tiab] OR reliab*[tiab] OR psychometr*[tiab] OR “cross-cultural”[tiab] OR adaptat*[tiab] OR translat*[tiab])) AND (“Feeding Behavior”[MeSH] OR “Eating”[MeSH] OR “Diet”[MeSH] OR “Nutrition Assessment”[MeSH] OR (“dietary behavior”[tiab] OR “eating behavior”[tiab] OR “food habit*”[tiab] OR “dietary habit*”[tiab] OR “nutritional habit*”[tiab] OR “dietary intake”[tiab] OR “food consumption”[tiab])) AND ((“Arabic version”[tiab] OR “Arabic translation”[tiab] OR “in Arabic”[tiab] OR “Arabic adaptation”[tiab]) OR ((“scale”[tiab] OR “questionnaire”[tiab] OR “survey”[tiab] OR “instrument”[tiab] OR “test”[tiab]) AND “Arabic”[tiab])) AND (Afghanistan[ad] OR Bahrain[ad] OR Djibouti[ad] OR Egypt[ad] OR Iran[ad] OR Iraq[ad] OR Jordan[ad] OR Kuwait[ad] OR Lebanon[ad] OR Libya[ad] OR Morocco[ad] OR Oman[ad] OR Pakistan[ad] OR Qatar[ad] OR “Saudi Arabia”[ad] OR Somalia[ad] OR Sudan[ad] OR Syria[ad] OR Tunisia[ad] OR “United Arab Emirates”[ad] OR Yemen[ad] OR Palestine[ad] OR Algeria[ad] OR “Middle East”[ad] OR “North Africa”[ad])).

Scopus: TITLE-ABS-KEY ((“dietary behavio*” OR “eating behavio*” OR “food habit*” OR “dietary habit*” OR “nutritional habit*” OR “food intake” OR “dietary intake” OR “nutritional intake” OR “diet quality” OR “food consumption” OR “eating pattern*” OR “dietary pattern*”) AND (validat* OR psychometric* OR reliab* OR “cross-cultural” OR adaptat* OR translat* OR “factor analysis” OR “cronbach’s alpha” OR “internal consistency” OR “test-retest”) AND (“Arabic version” OR “Arabic translation” OR “Arabic adaptation” OR “in Arabic” OR “Arabic scale” OR “Arabic questionnaire” OR “Arabic instrument” OR “Arabic validation”) AND (scale OR questionnaire OR survey OR instrument OR tool OR measure OR INDEX OR inventory OR assessment OR test) AND (Afghanistan OR Bahrain OR Djibouti OR Egypt OR Iran OR Iraq OR Jordan OR Kuwait OR Lebanon OR Libya OR Morocco OR Oman OR Pakistan OR Qatar OR “Saudi Arabia” OR Somalia OR Sudan OR Syria OR Tunisia OR “United Arab Emirates” OR Yemen OR Palestine OR Algeria OR “Middle East” OR “North Africa” OR “Arab world” OR “MENA” OR “GCC”)).

Web of Science: TS = ((scale OR questionnaire OR tool OR “assessment instrument” OR measure OR index OR inventory OR test) AND (“dietary behavior” OR “eating behavior” OR “food habits” OR “nutritional intake” OR “dietary intake” OR “food consumption”) AND (validation OR psychometric OR reliability OR validity OR “cross-cultural” OR adaptation OR translation) AND (“Arabic version” OR “Arabic translation” OR “in Arabic” OR “Arabic adaptation”) AND (Morocco OR Algeria OR Tunisia OR Egypt OR Jordan OR Lebanon OR Qatar OR Kuwait OR Oman OR Bahrain OR Iraq OR Syria OR Yemen OR Libya OR Sudan OR Palestine OR “Saudi Arabia” OR “United Arab Emirates” OR “North Africa” OR “Middle East”)).

In addition to the systematic search across electronic databases (PubMed, Scopus, and Web of Science), a complementary manual search was performed using Google Scholar and by screening the reference lists of the included studies. This process identified two additional articles that met the inclusion criteria.

### 2.4. Steps for Selection

**Figure 1:**
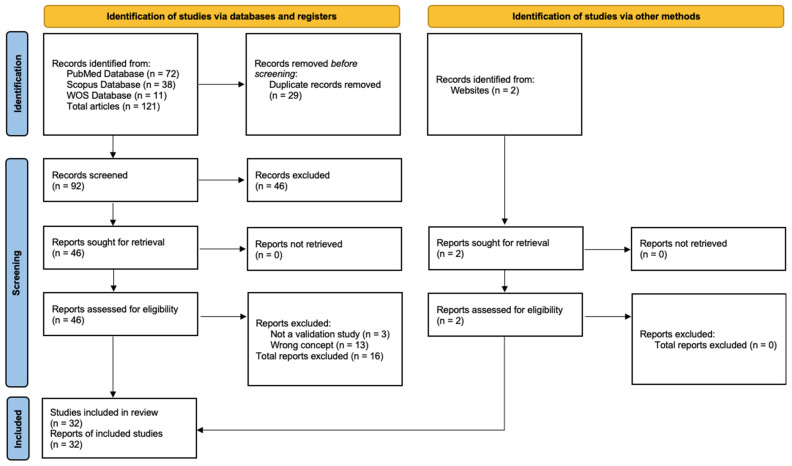
PRISMA flow diagram of study selection.

Two reviewers independently screened the titles and then abstracts of identified records for inclusion in the review. The full text of all records passing the title and abstract screening was retrieved. Two reviewers independently confirmed final eligibility. Discrepancies during the screening and full-text review phases were resolved through consensus or consultation with a third senior researcher. Figure 1 presents the PRISMA flowchart.

### 2.5. Data Extraction

Two reviewers extracted study details about the name of the instrument, the construct measured, the number of items and subscales, the country in which the validation was conducted, the original language of the tool, the target population, the sample size and the demographic characteristics of the participants (mean age, and percentage of men), the translation and cultural adaptation process, and the psychometric properties assessed (internal consistency, test–retest reliability, structural validity, construct validity, criterion validity, responsiveness, etc.). Information on the acceptability of the instrument and floor/ceiling effects were also collected when available. The extraction was performed independently by two evaluators.

Data extraction was performed in Microsoft Office Excel using a standardized data extraction form.

### 2.6. Quality Assessment

The methodological quality of each of the included studies was assessed in accordance with the COSMIN (COnsensus-based Standards for the selection of health Measurement Instruments) Risk of Bias checklist recommendations [10]. For each study, the various methodological domains of the COSMIN Risk of Bias checklist were examined, including the following:

- PROM development,
- Content validity,
- Structural validity,
- Internal consistency,
- Cross-cultural validity/measurement invariance,
- Reliability,
- Measurement error,
- Criterion validity,
- Hypotheses testing for construct validity,
- Responsiveness.

Each domain was evaluated using the scoring system recommended by COSMIN, classifying the quality of the methodology as “very good”, “adequate”, “doubtful”, “inadequate”, or “not applicable”.

The COSMIN Risk of Bias ratings are color-coded: very good (green), adequate (orange), doubtful (yellow), inadequate (red), and not applicable (white).

After this first stage dedicated to methodological quality, a second evaluation was carried out to assess the quality of the psychometric properties reported for each instrument.

In accordance with the COSMIN criteria for good measurement properties [11], each property was classified as follows:

- Satisfactory (+) when the results met the COSMIN criteria.
- Insufficient (−) when they did not meet the established standards.
- Undetermined (?) when the information was missing, incomplete, or the methodology used did not allow for a conclusion.

This dual approach, evaluating both the quality of the methodology and the quality of the psychometric properties, provided a rigorous and comprehensive analysis of the robustness of each measurement instrument validated in Arabic.

### 2.7. Ethical Considerations

This review focuses on published articles; therefore, ethical approval was not required.

## 3. Results

### 3.1. Study Selection

A total of 121 records were identified through database searching, including PubMed (*n* = 72), Scopus (*n* = 38), and Web of Science (*n* = 11). Additionally, two records were located through other sources. After removing duplicates, 94 records remained for screening [12,13].

Based on title and abstract screening, 46 records were excluded, as they did not meet the eligibility criteria. The remaining 48 full-text articles were assessed for eligibility. During this phase, 16 articles were excluded for a couple of reasons: three studies were not validation studies, and 13 were excluded due to focusing on the wrong concept.

Ultimately, 32 articles focusing on the development, cross-cultural adaptation, or validation of dietary behavior measurement scales in the Arabic language were included in the systematic review. Figure 1 presents the PRISMA flow diagram of the study selection process.

### 3.2. Study Characteristics

Publication dates spanned from 2006 to 2025, with a marked acceleration in research output; notably, 75% of the included studies were published after 2019. Geographically, the studies were conducted across nine countries in the MENA region. Lebanon contributed the highest number of studies (*n* = 14), followed by Saudi Arabia (*n* = 6). The remaining studies were distributed as follows: Kuwait (*n* = 2), Morocco (*n* = 2), Jordan (*n* = 2), Qatar (*n* = 2), United Arab Emirates (UAE) (*n* = 2), Oman (*n* = 1), and Egypt (*n* = 1).

The sample sizes varied widely, ranging from 50 participants in a Saudi Arabian study on sedentary behavior and dietary diet to 2312 schoolchildren in a Kuwaiti study on eating disorders. The median sample size was 363 participants. The age of participants across the included studies ranged from school-aged children (mean age ~10–11 years) to older adults (≥60 years). For studies targeting adult populations, the mean age predominantly fell between 20 and 30 years, reflecting the high proportion of university student samples.

This review identified three primary types of methodological focus among the 32 articles:

- Cross-cultural adaptation and validation: The vast majority of studies (*n* = 23) focused on translating an existing instrument (mostly from English) and validating its psychometric properties in an Arabic-speaking context.
- Development: A smaller subset of studies (seven studies) involved the development of new culture-specific instruments. For instance, the Food Frequency Questionnaires (FFQs) developed for Qatar and the UAE were designed specifically to capture local dietary habits rather than adapting Western tools.
- Translation/adaptation only: A few studies (two studies) focused primarily on the linguistic translation and cultural adaptation process with preliminary testing, rather than a full-scale psychometric validation.

Table 1 summarizes the characteristics of included studies.

### 3.3. Instrument Characteristics

A diverse range of constructs was measured by 32 instruments:

- Dietary intake (nine studies): Food Frequency Questionnaires (FFQs) were the most common, with specific versions for athletes, the elderly, and children.
- Diet quality and patterns (five studies): Instruments included the Mediterranean Diet Scale (MDS) and Mediterranean Diet Adherence Screener (MEDAS).
- Disordered eating (nine studies): A significant number of studies focused on Orthorexia Nervosa (ORTO-15, ORTO-R, TOS, and DOS). Other tools assessed Binge Eating (BES), Night Eating, and general eating psychopathology (EDE-Q, EDE-QS).
- Behavioral and psychological factors (nine studies): Additional constructs included Mindful Eating, Food–Mood associations, and Parental Feeding Practices.

The target populations were categorized as follows:

- General adults and university students: The most frequent population, including adults in Lebanon, Saudi Arabia, Morocco, and Oman.
- Children and adolescents: Seven studies focused specifically on these groups, validating tools for schoolchildren and adolescents in Lebanon, Kuwait, Jordan, and Saudi Arabia.
- Clinical and special populations: Studies also targeted specific groups such as athletes (Lebanon), pregnant women (Qatar), patients in cardiac rehabilitation (Saudi Arabia), patients post-bariatric surgery (Saudi Arabia), and children with Cerebral Palsy (Jordan).

Instrument complexity varied by construct: Item counts ranged from brief 7-to-10-item screening tools, such as the MEDAS, to comprehensive FFQs containing up to 160 items. Most behavioral scales, like Orthorexia or Body Image, contained between 10 and 28 items. Dimensionality: Several scales were validated as single-factor structures, particularly short screening tools (e.g., ORTO-R in some analyses). Meanwhile, complex constructs were often represented by multi-factorial models. For example, the Teruel Orthorexia Scale (TOS) typically exhibited a 2-factor structure (Healthy Orthorexia vs. Orthorexia Nervosa). Similarly, the Eating Disorders Examination Questionnaire (EDE-Q) and Mindful Eating Behavior Scale presented with three or four subscales covering distinct behavioral aspects (e.g., restraint, eating concern, and shape concern). Table 1 summarizes the instrument characteristics.

### 3.4. Psychometric Properties

Table 1 also presents the psychometric properties of the included instruments. The selected studies used various statistical methods to assess the reliability and validity of the Arabic versions of these instruments. The reporting of psychometric indicators varied depending on the nature of the instrument. Reliability was most frequently assessed through internal consistency and temporal stability. Internal consistency was frequently assessed, with most studies (*n* = 21) utilizing Cronbach’s alpha (α), which generally ranged from 0.70 to 0.94, indicating acceptable-to-excellent reliability. For instance, the Binge Eating Scale (BES) validations in Saudi Arabia and Lebanon reported high internal consistency (α = 0.88 and higher). Several recent studies, like the Saudi validation of the BES, also reported McDonald’s Omega (ω), a more robust estimate of reliability, which yielded comparable high values (ω = 0.88). Stability over time was assessed in a subset of studies using the Intraclass Correlation Coefficient (ICC). This was particularly prominent in the validation of Food Frequency Questionnaires (FFQs), such as the Moroccan study, which reported ICCs ranging from 0.69 to 0.84 for energy-adjusted nutrient intakes, indicating good reproducibility.

Construct validity was primarily established through factor analysis, differentiating between exploratory and confirmatory approaches. Exploratory factor analysis (EFA) was commonly used when the factor structure of the Arabic version was unknown or expected to differ from the original. Studies typically reported the Kaiser–Meyer–Olkin (KMO) measure of sampling adequacy and Bartlett’s test of sphericity to justify the analysis. Confirmatory factor analysis (CFA) was also widely used to verify the original factor structures. Studies reported various goodness-of-fit indices, including the Comparative Fit Index (CFI), the Goodness of Fit Index (GFI), and the Root Mean Square Error of Approximation (RMSEA). For example, the Saudi BES study reported a CFI of 0.904 and an RMSEA of 0.073, suggesting an acceptable model fit. Similarly, validation studies for the Orthorexia scales (ORTO-R and TOS) utilized CFA to confirm theoretical models (e.g., bi-dimensional structures).

### 3.5. Methodological Quality Assessment (COSMIN Risk of Bias)

The methodological quality of the included studies was appraised using the COSMIN Risk of Bias tool [10]. The analysis reveals heterogeneity in quality, varying by the psychometric properties evaluated and the type of instrument.

Structural validity and internal consistency: The internal structure of the instruments represented the highest-rated methodological domain.

Internal consistency: The majority of studies received a “very good” quality rating for this domain, including validations for the MEDAS, MEBS, MDS-A, TOS, ORTO-R, and BES. This indicates that reliability analyses (such as Cronbach’s alpha or Omega) were reported rigorously. Only a few studies, such as the NEQ validation, were rated as being “inadequate”.

Structural validity: The quality of factor structure evaluation was predominantly judged as “adequate” or “very good” (e.g., TOS, ORTO-R, ACAFSS, and EAT-26). However, some studies exhibited “doubtful” or “inadequate” quality (e.g., NEQ or the adolescent scale), often due to limitations in sample size relative to the number of items or the use of less robust factor analysis methods.

Content and cross-cultural validity: Content validity assessment emerged as a weakness in several studies, while cross-cultural adaptation showed variable results. Content validity: A significant number of studies received a “doubtful” rating, notably for the GALEN-FFQ, MEBS, ORTO-15, FMQ, and BES. This rating frequently reflects a lack of detail regarding the involvement of the target population during the item development or adaptation phase (e.g., qualitative or cognitive interviews). Conversely, studies such as those for the DHQ, EBBS, and ACAFSS were distinguished by “very good” quality. Cross-cultural validity: The methodological quality of cultural adaptation was rated as “Very Good” for instruments like the DHQ, EBBS, and A-PNAS, but remained “adequate” or not applicable (NA) for a large portion (87,5%) of the other works.

Reliability and criterion validity: These properties primarily concerned Food Frequency Questionnaires (FFQs) and specific scales. Test–retest reliability: When assessed (primarily for FFQs), the methodology was judged as “adequate” (e.g., GA^2^LEN-FFQ and MEDAS) or “very good” (DHQ II and A-PNAS).

Criterion validity: For instruments compared to a gold standard (such as 24 h recalls for FFQs), quality varied from “doubtful” (GALEN-FFQ and MEDAS) to “very good” (ACAFSS).

Hypothesis testing: Used for convergent validity, this domain generally received an “adequate” rating for most psychometric scales (MDS-A, TOS, ORTO-R, and BES).

Finally, measurement error was not evaluated in most included studies (87.5%). Meanwhile, responsiveness was not evaluated in any of the included studies (*n* = 0). Table 2 summarizes methodological quality assessment using COSMIN Risk of Bias of included studies.

### 3.6. Psychometric Properties Assessment

The psychometric properties assessment is detailed in Table 3. The evaluation of measurement properties for the 32 included instruments reveals a clear dichotomy in psychometric quality. Internal consistency and structural validity were the strongest properties, with approximately half of the instruments demonstrating sufficient evidence (+) for their factor structure and reliability. Cross-cultural validity was also frequently supported, indicating successful adaptation processes for the majority of the tools. However, significant gaps remain. Reliability (test–retest) yielded mixed results; while generally sufficient for Food Frequency Questionnaires, it was often found insufficient or not reported for behavioral scales. Notably, content validity was unreported for a large proportion of studies, suggesting a lack of qualitative validation. Furthermore, responsiveness and measurement error were almost universally absent (NA) across the entire sample, highlighting a critical lack of longitudinal evidence to support the use of these instruments for monitoring changes over time.

## 4. Discussion

The objective of this systematic review was to map and systematically evaluate all studies involving the translation, cultural adaptation, and/or validation of Arabic-language instruments measuring dietary behaviors in the Arab countries of the Middle East and North Africa (MENA) region. The 32 included studies presented instruments covering a wide range of concepts, notably diet adherence, dietary intake and nutritional quality, eating behaviors, and eating disorders, as well as context-specific behaviors such as child feeding practices, breastfeeding behavior, dietary behaviors during pregnancy, and eating after bariatric surgery. A notable 75% of included studies were published post-2019, reflecting a burgeoning research interest in dietary assessment and eating behaviors within the MENA region.

While research output in the region is accelerating, this quantitative growth is not matched by methodological rigor. Our evaluation highlights a stark reality: only 30% of the psychometric evaluations met the threshold for “adequate” or “very good” quality. Most critically, the fundamental ability of these instruments to detect responsiveness to change was entirely unaddressed across all 32 included studies. Furthermore, measurement error was neglected in 87.5% of the literature. These systemic omissions are not merely technical oversights; they signify that nearly all currently available Arabic dietary tools are incapable of reliably monitoring the success of nutritional interventions or public health policies.

Methodologically, one of the most striking findings of this review is the low quality of the instrument development processes and their cross-cultural adaptations. The vast majority (>85%) of the studies received an “indeterminate” (NA) or “doubtful” rating for development validity. This was primarily due to insufficient reporting of translation procedures, a lack of expert panel involvement, and the absence of cognitive interviews or pilot testing with the target population prior to psychometric testing.

Content validity, considered the most critical measurement property by COSMIN, was assessed in 87.5% of the studies (*n* = 28) and was frequently judged as methodologically doubtful or inadequate. It should be noted that the 87.5% refers to the methodological quality assessment of content validity (COSMIN Risk of Bias). By contrast, it evaluates the measurement property result according to the COSMIN criteria for good measurement properties; the 56.25% “not reported” indicates that these studies did not provide sufficient data to judge the sufficiency of content validity. When evaluated, the quality was often compromised by limited end-user involvement and insufficient exploration of cultural relevance during the development or adaptation phases. This deficiency suggests that many current instruments may lack ecological validity, potentially failing to encapsulate dietary behaviors as they are conceptualized and experienced within MENA-specific cultural frameworks [43,44].

Structural validity and internal consistency were more frequently evaluated (72% and 78%, respectively), yet the results remained inconsistent. Although approximately half of the included studies (50% for structural validity and 68% for internal consistency) demonstrated adequate properties, a significant portion relied on exploratory factor analysis alone, without confirmatory factor analysis, and utilized small sample sizes that did not meet COSMIN recommendations [10]. Furthermore, Cronbach’s alpha was often reported as the sole indicator of reliability, despite its known limitations when the dimensionality of the scale is uncertain.

Criterion validity, although rarely evaluated (25%), was consistently judged as adequate (nearly all of assessed studies) when reported. This could reflect a selective publication bias, where only favorable results are published, or the use of well-established reference methods (such as 24 h recalls) in this limited subset of studies. Nevertheless, relying solely on criterion validity is insufficient, as it does not compensate for poor content validity or unreliable measurement properties.

Test–retest reliability was evaluated in some of the studies (31%, *n* = 10) and was judged as adequate in all of the accessed studies. Even more concerning is the nearly complete absence (87.5%) of measurement error assessment across all included studies. Measurement error is essential for interpreting change scores and determining minimal important differences, particularly in intervention and longitudinal studies [45].

Responsiveness, defined as the ability of an instrument to detect meaningful changes over time, was not evaluated in all studies (100%). This gap seriously limits the utility of the instruments identified in this review for assessing nutritional interventions or policy-related changes in the MENA region. Similar inadequacies have been reported in systematic reviews of patient-reported outcome measures in other health fields, suggesting a generalized lack of methodological rigor rather than an isolated issue [46].

The methodological limitations observed in this review must be interpreted with caution due to the unique sociocultural dynamics of the MENA region. Dietary practices in this context are deeply shaped by religious and economic factors—such as communal eating, fasting periods like Ramadan, and traditional food habits—which may not be fully reflected in instruments originally developed in Western contexts [47]. Furthermore, the linguistic diversity of the region, which includes significant distinctions between Modern Standard Arabic and local dialects (e.g., Moroccan, Lebanese, or Saudi dialects), poses substantial challenges for the direct transfer of psychometric tools. The majority of the instruments included in this review were adaptations of tools originally developed in non-Arabic contexts (predominantly in English), and insufficient attention may have been paid to these cultural and linguistic nuances. This is evidenced by the finding that cross-cultural validity was either not reported or doubtful in over half of the studies (72%, *n* = 23). Consequently, certain items may lack relevance or be interpreted differently by respondents, potentially compromising the instrument’s accuracy. This risk is further underscored by the fact that content validity—the primary indicator of an instrument’s relevance—was absent or insufficient in 53% (*n* = 17) of the reviewed instruments, suggesting a critical gap in ensuring these tools are truly fit for the MENA population. Worryingly, only seven out of the 32 included studies explicitly reported whether they used Modern Standard Arabic (MSA) or a local dialect. The remaining studies simply referred to “Arabic” without further specification. This lack of transparency severely limits the ability to judge cross-cultural transferability. An instrument validated in Lebanese dialect cannot be assumed valid for a Moroccan population without additional linguistic and psychometric testing.

Our findings are consistent with previous systematic reviews conducted in non-Arabic contexts, which have also reported inadequate assessment of measurement error and responsiveness in dietary and behavioral instruments [48,49]. A review of eating behavior measures in Europe found that development validity and content validity were frequently inadequate or doubtful, and responsiveness was nearly always absent [50]. However, European validation studies more consistently reported structural validity using confirmatory factor analysis and larger sample sizes, whereas MENA studies more often relied on exploratory factor analysis alone. These disparities cannot be attributed solely to reporting omissions. Structural and systemic factors play a major role: research infrastructure in many MENA countries lacks dedicated psychometric support, advanced statistical software, or training in COSMIN guidelines; funding disproportionately favors epidemiological surveillance over methodological studies, leading to underfunded validation projects with small convenience samples (median 363 participants in this review); academic incentive structures prioritize publication quantity over rigor, making rapid adaptation of existing instruments more attractive than full COSMIN-compliant validation; the absence of validated Arabic versions of gold-standard reference instruments hampers criterion validity assessment; and limited multicenter collaborations perpetuate validation in single countries or dialects without testing measurement invariance across the Arab world. Addressing these systemic issues will require coordinated efforts from funders, journal editors, academic institutions, and regional research networks. Nevertheless, reporting omissions remain a concern: in this review, 78% of studies lacked a formal assessment of development validity, while content validity was entirely absent for 12% of the evaluated instruments.

Methodological disparities: In contrast to international validation studies that increasingly utilize confirmatory factor analysis and large multicentric samples, a significant number of MENA studies relied on smaller, monocentric samples (with a median sample size of 363 participants) and less rigorous validation strategies. These differences likely reflect disparities in research infrastructure, training in psychometrics, and access to methodological expertise, rather than a lack of scientific interest.

The findings of this review have important implications for researchers, clinicians, and policymakers in the MENA region. The widespread use of insufficiently validated instruments can lead to biased estimates of eating behaviors, misclassification of eating disorders, and erroneous conclusions about associations between diet and health outcomes. This is particularly problematic in a region facing a double burden of malnutrition and noncommunicable diseases [51].

Based on the methodological quality and the sufficiency of measurement properties, the following instruments are recommended per construct category, with the caveat that no instrument has been tested for responsiveness and very few have evaluated measurement error:

- Dietary intake: The Moroccan FFQ [14] has the strongest evidence (very good internal consistency and test–retest reliability; adequate criterion validity). The Qatari [29] and Lebanese older-adult FFQs [26] are acceptable, but with lower structural validity. No FFQ is recommended for children.
- Diet quality/Mediterranean diet adherence: The Arabic MDS [16] is more robust than the MEDAS [12], with very good structural validity and internal consistency.
- Disordered eating—adolescents: The TOS [17] is recommended for orthorexia (very good internal consistency and structural validity). The BES [36,39] is acceptable for binge eating, but with caution.
- Disordered eating—adults: For multi-country studies, the EDE-Q [42] is preferred due to cross-cultural validity. For adult orthorexia, the ORTO-R [18,38] is acceptable despite doubtful content validity.
- Mindful eating: Only the MEBS [15] exists; suitable for exploratory research only, not clinical decisions. Further qualitative validation is urgently needed.
- Parental feeding practices: The Arabic CFPQ [33] is adequate for cross-sectional studies, but not for longitudinal interventions.
- Other constructs (pregnancy and post-bariatric surgery): Promising, but require further validation.

This review has several strengths, including a comprehensive search strategy, adherence to PRISMA guidelines, and use of the COSMIN framework for assessing methodological quality. To our knowledge, this is the first systematic review focusing specifically on Arabic-language eating behavior measurement instruments in the MENA region.

However, certain limitations must be acknowledged. First, this review was limited to published studies, which may introduce publication bias; while we performed a comprehensive search of three major databases, we did not search the grey literature (e.g., theses, conference abstracts, and unpublished reports), which may contain validation studies with null or negative findings. This is particularly relevant for criterion validity, which was reported in only seven studies and was rated “adequate” in all of them. Such a pattern raises concern that studies showing poor criterion validity may not have been published, or that non-significant results were selectively omitted from published reports. Many journals preferentially accept validation studies that demonstrate good psychometric properties, potentially inflating the apparent quality of instruments. To mitigate this bias in the future, we encourage researchers to pre-register their validation protocols and to report all psychometric results, regardless of whether they meet a priori thresholds. Until such practices become routine, systematic reviews of measurement properties should interpret the frequency of “adequate” ratings with caution. Second, the heterogeneity of study designs and reports limited the possibility of quantitative synthesis. Finally, incomplete reporting in several studies made it difficult to fully assess certain psychometric properties, potentially leading to conservative quality assessments. Finally, A further practical consideration concerns intellectual property. Several instruments (e.g., EAT-26 and EDE-Q) are copyrighted; however, most copyright holders permit non-commercial academic use without fees. However, commercial use or inclusion in paid digital platforms requires explicit licensing agreements. For researchers in the MENA region with limited administrative or legal support, navigating these requirements can be challenging. We recommend that investigators always verify terms of use before translating or validating any copyrighted instrument and consider open-access alternatives when possible. Future validation studies should explicitly report any permissions obtained.

## 5. Conclusions

This systematic review highlights the increasing availability of Arabic-language instruments for measuring dietary behaviors, with a marked acceleration of research in the MENA region since 2020. However, critical appraisal of the 32 included studies indicates that this quantitative expansion of the literature is not consistently accompanied by requisite methodological rigor. While structural validity and internal consistency are generally well-established for the majority of scales, critical gaps persist in content validity, measurement error, and especially responsiveness.

To guide future research, we propose the following prioritized operational recommendations:

- Priority instrument categories for rigorous validation: Responsiveness and measurement error should be evaluated as a matter of urgency for the most widely used instruments, particularly Food Frequency Questionnaires (FFQs) and eating disorder scales (e.g., TOS, BES, and EDE-Q), given their frequent use in intervention studies. New validation studies should focus on mindful eating and parental feeding practices, where only single instruments exist with doubtful content validity.
- Underrepresented countries and populations: Validation studies are heavily concentrated in Lebanon and Saudi Arabia. Urgent research is needed in North African countries (e.g., Algeria, Libya, Tunisia, and Sudan), the Levant (e.g., Syria and Palestine), and the Gulf (e.g., Bahrain, Oman, and Yemen). Special populations remain neglected, including rural communities, low-literacy groups, pregnant women (only one study), and clinical populations with eating disorders.
- Methodological standards for future validations: Researchers should adhere to COSMIN guidelines, pre-register their protocols, explicitly report the language register (MSA vs. local dialect), and test measurement invariance across Arab countries. Multicenter collaborations within the MENA region are strongly encouraged to improve sample diversity and cross-cultural validity.

Until these gaps are addressed, existing instruments should be used with caution, particularly for monitoring change over time, and qualitative cognitive testing should complement quantitative validation in future studies.

## Figures and Tables

**Table 1:** General and psychometric characteristics of the included studies (*n* = 32).

First Author, Year, Country	Instrument Name	Study Design	Sample Size	Age, y (M ± SD)	Gender (Male %)	Recruitment Method	Target Population	Language of Administration	Original Language	Construct Measured	Number of Items (Subscales)	Reference Standard	Key Reliability Statistic	Key Validity Statistic
**El Kinany et al. [14], 2018, Morocco**	Adapted GA^2^LEN Food Frequency Questionnaire (FFQ)	Cross-sectional validation study	87	27.3 ± 5.7	29.9	Convenience sample (University Hospital staff)	Adults	Moroccan Arabic	English	Usual dietary intake	255 (32)	Mean of three 24 h dietary recall questionnaires	ICC ranged from 0.69 (fat) to 0.84 (Vitamin A) for energy-adjusted nutrient intakes (FFQ1 vs. FFQ2)	Correlations between FFQ1 and 24HRs ranged from 0.24 (fiber) to 0.93 (total MUFA)
**Sammoud et al. [12], 2023, Morocco**	Mediterranean Diet Adherence Screener (MEDAS)	Cross-sectional validation study	160	28.59 ± 7	33.1	Convenience sample (non-medical hospital staff)	Adults	Moroccan Arabic (Darija)	Spanish	Adherence to the Mediterranean Diet	14 (1)	MedQ-Sus	K-R21 = 0.85; ICC (1-week) = 0.88	Correlation with MedQ-Sus (rho = 0.49); AUC = 0.74
**Fekih-Romdhane et al. [15], 2023, Lebanon**	Mindful Eating Behavior Scale (MEBS)	Cross-sectional validation study	359	22.75 ± 7.04	40.1	Online convenience sample (snowball)	Arabic-speaking adults	Arabic (Modern Standard Arabic)	English	Mindful Eating Behavior	17 (4)	NR	ω = 0.82 − 0.95	CFA fit indices (CFI = 0.93); full metric and scalar invariance by sex
**Aljehani et al. [16], 2023, Saudi Arabia**	Mediterranean Diet Scale (MDS)	Cross-sectional validation study	200	46.5 ± 13.6	46.0	Convenience sample from a cardiology center	Arabic-speaking cardiac patients or those with cardiovascular risk factors	Arabic	German	Adherence to the Mediterranean Diet Pattern	13 (4)	Cardiac rehabilitation (CR) participation	α = 0.74	Significant association with CR participation (*p* = 0.002); correlations with clinical/socioeconomic variables
**Mhanna et al. [17], 2022, Lebanon**	Teruel Orthorexia Scale (TOS)	Cross-sectional validation study	555	16.67 ± 1.00	24.3	Snowball sampling (online)	Adolescents (15–18 years)	Arabic	Spanish	Orthorexia Nervosa (OrNe) and Healthy Orthorexia (HeOr)	17 (2)	DOS and ORTO-R scales	α (OrNe) = 0.853 α (HeOr) = 0.829	CFA: χ^2^ = 429.09, CFI = 0.954, RMSEA = 0.069; correlations with DOS (r = 0.715 OrNe, 0.754 HeOr) and ORTO-R (r = −0.437 OrNe, −0.305 HeOr)
**Rogoza et al. [18], 2022, Lebanon**	ORTO-R	Cross-sectional validation study	363	22.65 ± 3.48	38.3	Convenience sampling (university students; online survey)	Young adults (university students)	Arabic	English	Orthorexic behaviors	6 (1)	TOS, EAT-26, perfectionism scales, anxiety, depression, self-esteem scales	α = 0.78	CFA: χ^2^ = 23.53, CFI = 0.959, RMSEA = 0.081; correlations with TOS-OrNe (r = 0.51), EAT-26 (r = 0.40), depression (r = 0.41)
**Rogoza et al. [19], 2021, Lebanon**	Düsseldorf Orthorexia Scale (DOS)	Cross-sectional validation study	555	16.66 ± 1.01	24.3	Snowball sampling via online questionnaire	Adolescents (15–18 years)	Arabic	German	Orthorexic eating behavior (Orthorexia Nervosa)	10 (1)	TOS (Teruel Orthorexia Scale) and ORTO-R (for convergent validity)	α = 0.85	CFA (one-factor with residual correlation): χ^2^ = 163.39, CFI = 0.970, RMSEA = 0.079; correlations with TOS-OrNe (r = 0.715), TOS-HeOr (r = 0.754), ORTO-R (r = −0.383)
**Charafeddine et al. [20], 2020, Lebanon**	Breastfeeding Behavior Questionnaire (BBQ)	Instrument validation study (using secondary data from a clinical trial)	354	29.3 ± 4.9	0	Consecutive recruitment from outpatient obstetric clinics of two tertiary care centers	Pregnant women intending to breastfeed	Arabic	English	Women’s perceptions of their breastfeeding behavior	12 (1)	NR	α = 0.78	Correlations with IIFAS-A (r = −0.397), BFK-A (r = −0.161), IFI-A (r = −0.210)
**Alaqil et al. [8], 2023, Saudi Arabia**	Sedentary Behavior Questionnaire (SBQ); Dietary Habits Questionnaire (adapted from SHARE); Preclinical Mobility Limitation questionnaire	Cross-sectional study (translation and cultural adaptation)	50	41.7 ± 9.6	52	Convenience sampling via online flyers (Twitter and WhatsApp) and a research center	Adult population (>30 years)	Arabic	English	Sedentary behavior, Dietary habits, Preclinical mobility limitation	SBQ: 9; Dietary: 4; Mobility: 3	NR	NR	NR
**Alsehemi et al. [21], 2023, Saudi Arabia**	Arabic Eating Behavior after Bariatric Surgery (EBBS) questionnaire	Cross-sectional validation study	390	36.82 ± 9.67	43.8	Online self-reported survey distributed via social media (Twitter, Telegram, WhatsApp, and Facebook)	Patients who underwent metabolic and bariatric surgery ≥3 years prior	Arabic	English	Adherence to dietary and lifestyle recommendations post-bariatric surgery	10 (4)	NR	α = 0.851	Correlations with %TWL (r = 0.12 to 0.14) and %WLM (r = 0.18). EFA indicated non-unidimensional structure
**Alruwaitaa et al. [22], 2022, Saudi Arabia**	Adult Eating Behavior Questionnaire (AEBQ)	Cross-sectional validation study	576	35.61 ± 12.85	28	Online distribution via social media platforms	general adult population	Arabic	English	Appetitive traits (food approach and avoidance behaviors)	27 (6)	NR	α = 0.72	EFA resulted in a distinct 6-factor structure. Convergent validity: Positive correlation between Emotional Over-Eating (EOE) and BMI (r = 0.16, *p* < 0.01). Content validity: Scale Content Validity Index, average method (S-CVI/Ave) = 0.95
**Sannan et al. [23], 2024, Lebanon**	Food Frequency Questionnaire (FFQ) for athletes	Cross-sectional development and validation study	194	30.05	94.3	Professional athletes from Lebanese Army and Internal Security Forces sports teams	Lebanese athletes	Arabic	Arabic	Dietary intake	157	24 h dietary recalls (24 h DRs)	ICC for proteins = 0.92	Spearman’s correlation for energy intake vs. 24 h DRs = 0.95
**Begdache et al. [24], 2024, United Arab Emirates (UAE)**	Food-Mood Questionnaire (FMQ)	Cross-sectional validation study	224	20.7 ± 4.1	27.2	Online convenience sample	College students	Arabic	English	Non-specific psychological distress and dietary intake	22 (3)	NR	α = 0.865 (Mental distress), 0.716 (Prudent diet), 0.531 (Western diet); ICC:0.667–0.866	EFA/CFA supported three-factor structure (χ^2^/df = 2.28, RMSEA = 0.076). Significant association between FMQ Western diet subscale and perceived unhealthy diet (β = 2.43, *p* < 0.05).
**Haddad et al. [25], 2020, Lebanon**	ORTO-15	Cross-sectional validation study	806	27.59 ± 11.76	22.7	Proportionate random sample from households in selected villages across Lebanese governorates	General adult population	Arabic	Italian	Orthorexia Nervosa (ON) tendencies and behaviors	15 (3)	NR	α = 0.829 (total scale); α = 0.707, 0.751, 0.759 for the three subscales	EFA (KMO = 0.830) and CFA supported a three-factor structure (GFI = 0.964, AGFI = 0.919, RMSEA = 0.098). Significant negative correlations with EAT-26 (r = −0.326), DRES (r = −0.197), and body dissatisfaction (r = −0.143)
**Yaghi et al. [26], 2022, Lebanon**	Food Frequency Questionnaire (FFQ) for community-dwelling older adults	Cross-sectional development and validation study	76	74.4 ± 6.9	36.8	Random selection from a cross-sectional study	Community-dwelling adults aged 60 years and older	Arabic	Arabic	Habitual nutrient and dietary intake	90	Mean of two 24 h dietary recalls (24HDR)	(ICC) for calories = 0.93	Percentage agreement (same/adjacent quartile) for nutrient intake = 80%; energy-adjusted correlation for carbohydrates = 0.72
**Al-Farhan et al. [27], 2021, Kuwait**	Block Kids Food Frequency Questionnaire (FFQ)	Cross-sectional validation study	51	10.4 ± 0.4	47	School-based sample from 16 sex-specific schools in six cities	Fifth-grade children	Arabic	English	Habitual dietary intake	72	NR	ICC (1 week for boys, 4 weeks for girls). Food groups ICC = 0.68, Macronutrients ICC = 0.43, Selected Micronutrients ICC = 0.54	NR
**Al Uraimi et al., 2025 [13], Oman**	Omani Food Frequency Questionnaire (OFFQ) (adapted from DHQ II)	Cross-sectional development and validation study	62	22.47	48.4	Convenience sample of students and employees at university	Omani adults (healthy)	Arabic	English	Habitual dietary intake	415	NR	Median ICC (frequency): 0.569–0.807; Median ICC (portion): 0.395–0.821; Median weighted Kappa (frequency): 0.379–0.601; Median weighted Kappa (portion): 0.259–0.580; Cronbach’s α for food groups (frequency): 0.267–0.871	NR
**Almasri et al. [28], 2021, Jordan**	Arabic Parent Nutritional Assessment Scale (A-PNAS)	Cross-sectional development and validation study	130 (CP group) + 130 (typical development group)	Children with CP: 4.26 ± 3.29; Children with TD: 4.65 ± 3.54	Children with CP: 56.2; Children with TD: 60	Convenience sample from the national CP registry. Matched group of siblings/relatives	Parents/caregivers of children with cerebral palsy (and typical development, for comparison)	Arabic	Arabic	Parental-reported nutritional problems (food intake, health, and behavioral aspects) in children with CP	28 (3)	Known-groups validity (comparison with a matched group of children with TD)	α: Food Intake = 0.61, Health = 0.67, Behavioral = 0.38. ICCs (2–3-week interval): Food Intake = 0.96, Health = 0.98, Behavioral = 0.96	Factor analysis (EFA) variance explained: 31.6%. Known-groups discriminant validity (*p* < 0.001 for all subscales)
**Bawadi et al. [29], 2021, Qatar**	Culture-specific Food Frequency Questionnaire (FFQ) for Qataris. adapted from DHQ II	Cross-sectional development and validation study	107	33	37.4	Convenience sample of family members of Qatar University students	Healthy Qatari adults	Arabic	English	Habitual dietary intake	Modified DHQ II, initial list of 153 items; number after adaptation NR	Average of three 24 h dietary recalls (24 h DR)	Test–retest (1-month) Pearson correlation (FFQ1 vs. FFQ2) for macronutrients: r = 0.799 (average)	Validity correlation (FFQ2 vs. 24hDR) for macronutrients: r = 0.545–0.974 (except trans-fat). Quartile agreement rates (FFQ2 vs. 24hDR) for macronutrients: 71–100%
**Jamaluddine et al. [30], 2019, Lebanon**	Child-Reported Food Security Scale	Cross-sectional development and validation study (embedded in an intervention study)	1287	5–15	NR	School-based recruitment from UNRWA schools in Palestinian refugee camps	School-aged children (5–15 years) in refugee settings	Arabic (Palestinian dialect)	Items derived from the English literature	Child-reported food insecurity experience	10 (1)	Arab Family Food Security Scale (AFFSS—household level)	α = 0.89	Strong association with household food insecurity (OR: 2.3, *p* < 0.001)
**Alzaben et al. [31], 2025, Saudi Arabia**	Short Healthy Eating Index (sHEI)	Cross-sectional validation study	615	23.0 ± 7.6	11.4	Convenience sample of university students	Young Saudi adults	Arabic	English	Dietary Quality	22 (4)	NR	α for adequacy items = 0.81; for moderation items = 0.44	Item Content Validity Index (I-CVI) range: 0.89–0.99; KMO = 0.84; PCA explained 56.7% variance
**Elsadek et al. [32], 2014, Egypt**	Night Eating Questionnaire (NEQ)	Cross-sectional validation study	420	20.2	29	Convenience sample of university students	Young adult university students	Arabic	English	Symptoms of Night Eating Syndrome (Nocturnal Ingestions, Evening Hyperphagia, Morning Anorexia, Insomnia, Mood)	14 (5)	NR	α = 0.54	Factor loadings from confirmatory factor analysis ranged from 0.581 to 0.892
**Al-Qerem et al. [33], 2017, Jordan**	Comprehensive Feeding Practices Questionnaire (CFPQ)	Cross-sectional validation study	970	9.1	50.3	Convenience sample from five primary schools	Mothers of school-aged children (6–12 years)	Arabic	English	Parental (maternal) feeding practices	43 (11)	NR	α ranged from 0.66 to 0.90 across subscales	Confirmatory factor analysis fit indices: CMIN/DF = 2.18, CFI = 0.93, TLI = 0.92, RMSEA = 0.03
**Itani et al. [34], 2017, Lebanon**	Questionnaire to assess psychosocial determinants of eating behavior	Cross-sectional development and validation study	159	17–18	NR	Random sample from public schools	Arabic-speaking adolescents	Arabic	English	Psychosocial determinants of eating behavior (Knowledge, Attitude, Practices, Social Norms, Self-efficacy)	93 (5)	NR	α for total scales: 0.759–0.836. ICC ranged from 0.752 to 0.921	Content Validity Ratio (CVR) ≥ 0.778; Scale Content Validity Index (S-CVI) ≥ 0.934; exploratory factor analysis used.
**Honkala et al. [35], 2006, Kuwait**	Health Behavior in School-Aged Children (HBSC)	Cross-sectional study with translation and cultural adaptation	2312	11.9 ± 1.3	51	Two-stage random sampling: random selection of schools, then convenience sampling of 2–3 classes per school	Schoolchildren (ages 11–13)	Arabic	English	Health behaviors (incl. sugar consumption frequency)	84	NR	NR	NR
**Mina et al. [36], 2021, Lebanon**	Binge Eating Scale (BES)	Cross-sectional validation study	555	16.67 ± 1.00	24.3	Snowball sampling via online questionnaire	Lebanese adolescents (15–18 years)	Arabic	English	Binge eating behavior	16 (1)	NR	α = 0.835	CFA: One-factor model fit indices (CFI = 0.964, RMSEA = 0.041). Convergent validity via correlation with body dissatisfaction (r = 0.35), depression (r = 0.33), and self-esteem (r = −0.36)
**Kfoury et al. [37], 2025, Lebanon**	Eating Disorders Examination Questionnaire-Short Parent Version (EDE-QS-P)	Cross-sectional validation study	502	36.24 ± 8.29	25.5	Online snowball sampling	Lebanese parents of children aged 6–11 years	Arabic	English	Parent-reported disordered eating symptoms in children	11 (1)	NR	α = 0.96 ω = 0.96	CFA fit for one-factor model: χ^2^/df = 4.82; RMSEA = 0.087; CFI = 0.965; TLI = 0.955. AVE = 0.66. Full measurement invariance across parent sex (ΔCFI ≤ 0.001)
**Hallit et al. [38], 2021, Lebanon**	ORTO-R (Revised version of ORTO-15)	Cross-sectional validation study	783	27.78 ± 11.60	66.5	Proportionate random sampling from all Lebanese governorates (door-to-door in selected villages)	General adult population	Arabic	English	Orthorexia Nervosa (ON) thoughts and behaviors	6 (2)	NR	α = 0.755	CFA fit for two-factor model: χ^2^/df = 3.36; RMSEA = 0.077; CFI = 0.967; TLI = 0.914. Correlation with EAT-26: r = 0.428 (females) and 0.360 (males), *p* < 0.001
**Zeidan et al. [39], 2019, Lebanon**	Binge Eating Scale (BES)	Cross-sectional validation study	811	27.59 ± 11.76	33.5	Proportionate random sampling from all Lebanese governorates (door-to-door in selected villages)	General adult population	Arabic (native language of Lebanon)	English	Binge eating pathology	16 (2)	NR	α (total scale) = 0.862 α (Factor 1) = 0.826; α (Factor 2) = 0.682	CFA fit indices: χ^2^/df = 2.4; RMSEA = 0.12; GFI = 0.799; AGFI = 0.706. Correlations (with HAMD: r = 0.399, *p* < 0.001)
**Alyami et al. [40], 2024, Saudi Arabia**	Eating Attitudes Test (EAT-26)	Cross-sectional validation study	1734	26.88 ± 9.13	21.6	Convenience and snowball sampling (online social media)	General adult population	Arabic	English	Disordered eating behaviors and attitudes	26 (4)	NR	α = 0.88 ω = 0.88	CFA (16-item, 4-factor model): CFI = 0.904, SRMR = 0.0554, RMSEA = 0.073. Invariance (MGCFA): Configural, metric, scalar invariance supported across sex and BMI categories (ΔCFI ≤ 0.001)
**Silang et al. [41], 2025, Qatar**	Prenatal Eating Behaviors Screening Tool (PEBS)	Cross-sectional validation study	116	30.4 ± 4.9	0	Convenience sampling (tertiary hospital antenatal units)	Pregnant women (third trimester)	Modern Standard Arabic (MSA)	English	Prenatal eating behaviors / risk for eating disorders	12 (2)	NR	α = 0.77	EFA (2-factor): Better fit than single factor (AIC = 424.26). CFA (single factor): Most items’ loading > 0.65
**el Khazen Hadati et al. [42], 2024, United Arab Emirates (UAE)**	Eating Disorder Examination Questionnaire (EDE-Q)	Cross-sectional validation study	260	NR	13.1	Convenience sampling (clinical: outpatient clinic; non-clinical: community settings)	Arabic-speaking clinical population with eating disorders and general population	Modern Standard Arabic (MSA)	English	Eating disorder psychopathology	28	NR	ω = 0.919	CFA (7-item EDE-Q): CFI = 0.985, RMSEA = 0.042. Spearman’s rho between EDE-Q and CIA subscales = 0.59 to 0.86

**Table 2:** Methodological quality assessment of included studies (COSMIN Risk of Bias).

Instrument Name	Development	Content Validity	Structural Validity	Internal Consistency	Cross-Cultural Validity	Test–Retest Reliability	Measurement Error	Hypothesis Testing	Criterion Validity	Responsiveness
GA^2^LEN-FFQ [14]	⚪️	🟡	⚪️	⚪️	⚪️	🟠	⚪️	⚪️	🟡	⚪️
MEDAS [12]	⚪️	🟠	⚪️	🟢	⚪️	🟠	⚪️	🟡	⚪️	⚪️
MEBS [15]	⚪️	🟡	🟠	🟢	🟠	⚪️	⚪️	⚪️	🟠	⚪️
MDS-A [16]	⚪️	🟠	🟠	🟢	⚪️	⚪️	⚪️	🟠	🟠	⚪️
TOS [17]	⚪️	🟠	🟢	🟢	🟠	⚪️	⚪️	⚪️	🟠	⚪️
ORTO-R [18]	⚪️	🟠	🟢	🟢	⚪️	⚪️	⚪️	⚪️	🟠	⚪️
DOS [19]	⚪️	🟠	🟠	🟢	⚪️	⚪️	⚪️	⚪️	🟠	⚪️
BBQ [20]	⚪️	🟠	🟠	🟠	⚪️	⚪️	⚪️	⚪️	🟠	⚪️
DHQ [8]	⚪️	🟢	⚪️	⚪️	🟢	⚪️	⚪️	⚪️	⚪️	⚪️
EBBS [21]	⚪️	🟢	🟠	🟢	🟢	⚪️	⚪️	⚪️	🟠	⚪️
AEBQ [22]	⚪️	🟢	🟡	🟠	⚪️	⚪️	⚪️	⚪️	🟠	⚪️
FFQ [23]	🟡	⚪️	⚪️	⚪️	⚪️	🟠	🟠	🟢	🟠	⚪️
FMQ [24]	⚪️	🟡	🟡	🟠	⚪️	🟠	⚪️	🟡	🟠	⚪️
ORTO-15 [25]	⚪️	🟡	🟠	🟢	⚪️	⚪️	⚪️	⚪️	🟠	⚪️
FFQ [26]	🟡	⚪️	⚪️	⚪️	⚪️	🟠	🟠	🟢	🟠	⚪️
Block FFQ [27]	⚪️	🟡	⚪️	⚪️	⚪️	🟠	🟠	⚪️	⚪️	⚪️
DHQ II [13]	🟢	🟢	⚪️	🟡	🟠	🟢	⚪️	⚪️	⚪️	⚪️
A-PNAS [28]	🟠	🟢	🟡	🟡	🟠	🟢	🟢	⚪️	🟢	⚪️
DHQ II [29]	🟡	⚪️	⚪️	⚪️	🟡	🟠	⚪️	🟡	⚪️	⚪️
ACAFSS [30]	🟢	🟢	🟢	🟢	🟠	⚪️	⚪️	🟢	🟢	⚪️
sHEI [31]	⚪️	🟢	🟡	🟠	⚪️	⚪️	⚪️	⚪️	⚪️	⚪️
NEQ [32]	⚪️	🔴	🔴	🔴	⚪️	⚪️	⚪️	⚪️	⚪️	⚪️
CFPQ [33]	⚪️	🟡	🟠	🟠	⚪️	⚪️	⚪️	⚪️	🟠	⚪️
Adolescents EB Scale [34]	🟡	🟡	🔴	🟠	⚪️	🟠	⚪️	⚪️	⚪️	⚪️
HBSC [35]	⚪️	🟠	⚪️	⚪️	⚪️	⚪️	⚪️	⚪️	🟠	⚪️
BES [36]	⚪️	🟡	🟠	🟢	⚪️	⚪️	⚪️	⚪️	🟠	⚪️
EDE-QS-P [37]	⚪️	🟠	🟠	🟢	🟢	⚪️	⚪️	⚪️	🟠	⚪️
ORTO-R [38]	⚪️	🟡	🟠	🟢	🟠	⚪️	⚪️	⚪️	🟠	⚪️
BES [39]	⚪️	🟡	🟠	🟢	⚪️	⚪️	⚪️	⚪️	🟠	⚪️
EAT-26 [40]	⚪️	⚪️	🟢	🟢	🟠	⚪️	⚪️	⚪️	🟠	⚪️
PEBS [41]	⚪️	🟡	🟠	🟠	⚪️	⚪️	⚪️	⚪️	🟠	⚪️
EDE-Q [42]	⚪️	🟡	🟠	🟢	⚪️	⚪️	⚪️	⚪️	🟠	⚪️

🟢 Very good; 🟠 adequate; 🟡 doubtful; 🔴 inadequate; ⚪️ not applied.

**Table 3:** Summary of measurement properties frequencies (*n* =32).

Measurement Property	Sufficient (+)	Insufficient (−)	Indeterminate (±)	Not Reported (NA)
Content Validity	10	4	0	18
Structural Validity	15	3	3	11
Internal Consistency	16	3	4	9
Cross-Cultural Validity	15	4	4	9
Reliability (Test–Retest)	14	13	1	4
Measurement Error	0	0	0	32
Hypothesis Testing	15	2	3	12
Criterion Validity	7	0	0	25
Responsiveness	0	0	0	32

## Data Availability

No new data were created or analyzed in this study. Data sharing is not applicable to this article.

## Supplementary Materials

The following supporting information can be downloaded at https://www.mdpi.com/article/10.3390/nu18142343/s1, PRISMA 2020 Checklist. Ref. [52] was cited in the Supplementary Materials.

## Abbreviations

The following abbreviations are used in this manuscript:

APCs	Article processing charges
COSMIN	COnsensus-based Standards for the selection of health Measurement INstruments
DAAD	Deutscher Akademischer Austauschdienst
MENA	Middle East and North Africa
NA	Not applied
NR	Not reported
PROM	Patient-reported outcome measure

## References

[1] Neufeld L.M., Hendriks S., Hugas M., Von Braun J., Afsana K., Fresco L.O., Hassan M.H.A. (2023). Healthy Diet: A Definition for the United Nations Food Systems Summit 2021. Science and Innovations for Food Systems Transformation.

[2] The Role of Dietary Lifestyle Modification in Chronic Disease Prevention and Management—StatPearls—NCBI Bookshelf. https://www.ncbi.nlm.nih.gov/books/NBK587401/.

[3] Ma H., Wang M., Qin C., Shi Y., Mandizadza O.O., Ni H., Ji C. (2025). Trends in the burden of chronic diseases attributable to diet-related risk factors from 1990 to 2021 and the global projections through 2030: A population-based study. Front. Nutr..

[4] WHO EMRO—Unhealthy Diet. https://www.emro.who.int/noncommunicable-diseases/causes/unhealthy-diets.html.

[5] Musaiger A.O., Al-Hazzaa H.M. (2012). Prevalence and risk factors associated with nutrition-related noncommunicable diseases in the Eastern Mediterranean region. Int. J. Gen. Med..

[6] Curbing the Noncommunicable Disease Epidemic in the Middle East and North Africa. PRB. https://www.prb.org/resources/curbing-the-noncommunicable-disease-epidemic-in-the-middle-east-and-north-africa/.

[7] Bailey R.L. (2021). Overview of Dietary Assessment Methods for Measuring Intakes of Foods, Beverages, and Dietary Supplements in Research Studies. Curr. Opin. Biotechnol..

[8] Alaqil A.I., Gupta N., Alothman S.A., Al-Hazzaa H.M., Stamatakis E., Del Pozo Cruz B. (2023). Arabic translation and cultural adaptation of sedentary behavior, dietary habits, and preclinical mobility limitation questionnaires: A cognitive interview study. PLoS ONE.

[9] Ortiz-Gutiérrez S., Cruz-Avelar A. (2018). Translation and Cross-Cultural Adaptation of Health Assessment Tools. Actas Dermo-Sifiliográficas (Engl. Ed.).

[10] COSMIN (2018). Risk of Bias Checklist for Systematic Reviews of Patient-Reported Outcome Measures. Qual. Life Res..

[11] Mokkink L.B., Elsman E.B., Terwee C.B. (2024). COSMIN guideline for systematic reviews of patient-reported outcome measures version 2.0. Qual. Life Res..

[12] Sammoud K., Mahdi Z., Benzaida K., Elrhaffouli Y., Yamlahi M., Gourinda A., Charif F., Bousgheiri F., Elbouri H., Adil N. (2023). Cross-Cultural Adaptation of Mediterranean Diet Adherence Screener (MEDAS) Into Moroccan Arabic to Measure the Degree of Mediterranean Diet Adherence. Cureus.

[13] Al Uraimi T., Al Subhi L.K., Waly M., Al Rizeiqi M., Al Balushi R., Al Kharusi A. (2025). Development, Validity, and Reliability of a Food Frequency Questionnaire for Omani Adults. Nutrients.

[14] El Kinany K., Garcia-Larsen V., Khalis M., Deoula M.M.S., Benslimane A., Ibrahim A., Benjelloun M.C., El Rhazi K. (2018). Adaptation and validation of a food frequency questionnaire (FFQ) to assess dietary intake in Moroccan adults. Nutr. J..

[15] Fekih-Romdhane F., Malaeb D., Fawaz M., Chammas N., Soufia M., Obeid S., Hallit S. (2023). Translation and validation of the mindful eating behaviour scale in the Arabic language. BMC Psychiatry.

[16] Aljehani R., Aljehani G., Alharazi H., Horta P.M., Kümmel Duarte C., Ghisi G.L.D.M. (2023). The Mediterranean Diet Scale (MDS): Translation and validation of the Arabic version. PLoS ONE.

[17] Mhanna M., Azzi R., Hallit S., Obeid S., Soufia M. (2022). Validation of the Arabic version of the Teruel Orthorexia Scale (TOS) among Lebanese adolescents. Eat. Weight Disord..

[18] Rogoza R., Mhanna M., Gerges S., Donini L.M., Obeid S., Hallit S. (2022). Validation of the Arabic version of the ORTO-R among a sample of Lebanese young adults. Eat. Weight Disord..

[19] Rogoza R., Hallit S., Soufia M., Barthels F., Obeid S. (2021). Validation of the Arabic version of the Dusseldorf Orthorexia Scale (DOS) among Lebanese adolescents. J. Eat. Disord..

[20] Charafeddine L., Masri S., Shamsedine L., Ghandour L., Tamim H., El Khoury N., Hachem Z., Nabulsi M. (2020). Validation of the Arabic version of the breastfeeding behavior questionnaire among Lebanese women. Int. Breastfeed. J..

[21] Alsehemi N.H., Alharbi A.A., Alamri R.S., Fatani B.A., Alsenan S.H., Elbarazi I., Aldhwayan M.M. (2023). Translation and Validation of the Arabic Version of the Eating Behavior After Bariatric Surgery (EBBS) Questionnaire. Obes. Surg..

[22] Alruwaitaa M.A., Alshathri A., Alajllan L., Alshahrani N., Alotaibi W., Elbarazi I., Aldhwayan M.M. (2022). The Arabic Version of the Adult Eating Behavior Questionnaire among Saudi Population: Translation and Validation. Nutrients.

[23] Sannan N., Papazian T., Issa Z., El Helou N. (2024). Validity and reproducibility of a food frequency questionnaire to determine dietary intakes among Lebanese athletes. PLoS ONE.

[24] Begdache L., Radwan H., Abu Qiyas S., Abbas N., Naja F. (2024). Validity and Reliability of the Transcultural Arabic Adaptation of the Food-Mood Questionnaire Among College Students. Int. J. Environ. Res. Public Health.

[25] Haddad C., Hallit R., Akel M., Honein K., Akiki M., Kheir N., Obeid S., Hallit S. (2020). Validation of the Arabic version of the ORTO-15 questionnaire in a sample of the Lebanese population. Eat. Weight Disord..

[26] Yaghi N., Boulos C., Baddoura R., Abifadel M., Yaghi C. (2022). Validity and reliability of a food frequency questionnaire for community dwelling older adults in a Mediterranean country: Lebanon. Nutr. J..

[27] Al-Farhan A.K., Becker T.B., Petushek E., Weatherspoon L., Carlson J.J. (2021). Reliability of the Block Kid’s Food Frequency Questionnaire translated to Arabic and adapted for Kuwaiti children. Nutrition.

[28] Almasri N.A., Dunst C.J., Saleh M., Tayyem R. (2021). Development and Psychometric Properties of the Arabic Parent Nutritional Assessment Scale (A-PNAS) for Children with Developmental Disabilities. Phys. Occup. Ther. Pediatr..

[29] Bawadi H., Akasheh R.T., Kerkadi A., Haydar S., Tayyem R., Shi Z. (2021). Validity and Reproducibility of a Food Frequency Questionnaire to Assess Macro and Micro-Nutrient Intake among a Convenience Cohort of Healthy Adult Qataris. Nutrients.

[30] Jamaluddine Z., Sahyoun N.R., Choufani J., Sassine A.J., Ghattas H. (2019). Child-Reported Food Insecurity Is Negatively Associated with Household Food Security, Socioeconomic Status, Diet Diversity, and School Performance among Children Attending UN Relief and Works Agency for Palestine Refugees Schools in Lebanon. J. Nutr..

[31] Alzaben A.S., Bawazeer N.M. (2025). Translation, validity, and reliability of an Arabic version of the short Healthy Eating Index tested in young Saudi adults: A questionnaire-based study. Front. Public Health.

[32] Elsadek A.M., Hamid M.S., Allison K.C. (2014). Psychometric characteristics of the Night Eating Questionnaire in a Middle East population. Int. J. Eat. Disord..

[33] Al-Qerem W.A., Ling J., AlBawab A.Q. (2017). Validation of the comprehensive feeding practice questionnaire among school aged children in Jordan: A factor analysis study. Int. J. Behav. Nutr. Phys. Act..

[34] Itani L., Chatila H., Dimassi H., El Sahn F. (2017). Development and validation of an Arabic questionnaire to assess psychosocial determinants of eating behavior among adolescents: A cross-sectional study. J. Health Popul. Nutr..

[35] Honkala S., Honkala E., Al-Sahli N. (2006). Consumption of sugar products and associated life- and school-satisfaction and self-esteem factors among schoolchildren in Kuwait. Acta Odontol. Scand..

[36] Mina A., Hallit S., Rogoza R., Obeid S., Soufia M. (2021). Binge eating behavior in a sample of Lebanese Adolescents: Correlates and Binge Eating Scale validation. J. Eat. Disord..

[37] Kfoury M., Noureddine A., Malaeb D., Schuch F.B., El Khatib S., Dabbous M., Sakr F., Fekih-Romdhane F., Hallit S., Obeid S. (2025). Relationship between parental perfectionism and child’s disordered eating: Mediating role of parental distress and validation of the arabic version of the eating disorders examination questionnaire-short-parent version (EDE-QS-P). BMC Psychiatry.

[38] Hallit S., Brytek-Matera A., Obeid S. (2021). Orthorexia Nervosa and Disordered Eating Attitudes Among Lebanese Adults: Assessing Psychometric Proprieties of the ORTO-R in a Population-Based Sample. PLoS ONE.

[39] Zeidan R.K., Haddad C., Hallit R., Akel M., Honein K., Akiki M., Kheir N., Hallit S., Obeid S. (2019). Validation of the Arabic version of the binge eating scale and correlates of binge eating disorder among a sample of the Lebanese population. J. Eat. Disord..

[40] Alyami M.M., Al-Dossary S.A. (2024). Assessing disordered eating behaviours and attitudes: Factor structure and measurement invariance of the Arabic version of the eating attitudes test (EAT-26) in Saudi Arabia. J. Eat. Disord..

[41] Silang J.P.B., Minisha F., A-Hajjaji H.Q., Lim C.M., Thankappan J., Ali B.B.I.B., Prakash M.M., Mohamed Z.A., Mathew R., De Jesus D.H. (2025). Arabic translation and psychometric testing of the prenatal eating behaviors screening tool. J. Eat. Disord..

[42] El Khazen Hadati C., AKassie S., Bertl B., Fleifel Sidani M., Atef Wadiy Melad M., Ammar A. (2024). Psychometric Properties of the Eating Disorder Examination Questionnaire (EDE-Q) and the Clinical Impairment Assessment (CIA) Using a Heterogenous Clinical Sample from Arab Countries. Sage Open.

[43] Beaton D.E., Bombardier C., Guillemin F., Ferraz M.B. (2000). Guidelines for the Process of Cross-Cultural Adaptation of Self-Report Measures. Spine.

[44] Wild D., Grove A., Martin M., Eremenco S., McElroy S., Verjee-Lorenz A., Erikson P. (2005). Principles of Good Practice for the Translation and Cultural Adaptation Process for Patient-Reported Outcomes (PRO) Measures: Report of the ISPOR Task Force for Translation and Cultural Adaptation. Value Health.

[45] De Vet H.C., Terwee C.B., Mokkink L.B., Knol D.L. (2011). Measurement in Medicine: A Practical Guide.

[46] Terwee C.B., Mokkink L.B., Knol D.L., Ostelo R.W.J.G., Bouter L.M., de Vet H.C.W. (2012). Rating the methodological quality in systematic reviews of studies on measurement properties: A scoring system for the COSMIN checklist. Qual. Life Res..

[47] Musaiger A.O. (2011). Food Consumption Patterns in the Eastern Mediterranean Region.

[48] Burrows T.L., Martin R.J., Collins C.E. (2010). A systematic Review of the Validity of Dietary Assessment Methods in Children When Compared with the Method of Doubly Labeled Water. J. Am. Diet. Assoc..

[49] Lytle L.A., Nichaman M.Z., Obarzanek E., Glovsky E., Montgomery D., Nicklas T., Zive M., Feldman H., The CATCH Collaborative Group (1993). Validation of 24-hour recalls assisted by food records in third-grade children. J. Am. Diet. Assoc..

[50] Mokkink L.B., Prinsen C.A.C., Patrick D.L., Alonso J., Bouter L.M., de Vet H.C.W., Terwee C.B. (2018). COSMIN Methodology for Systematic Reviews of Patient-Reported Outcome Measures (PROMs)—User Manual.

[51] World Health Organization (2019). Strategy on Nutrition for the Eastern Mediterranean Region 2020–2030.

[52] Page M.J., McKenzie J.E., Bossuyt P.M., Boutron I., Hoffmann T.C., Mulrow C.D., Shamseer L., Tetzlaff J.M., Akl E.A., Brennan S.E. (2021). The PRISMA 2020 statement: An updated guideline for reporting systematic reviews. BMJ.

